# Kaempferol and Kaempferol Rhamnosides with Depigmenting and Anti-Inflammatory Properties

**DOI:** 10.3390/molecules16043338

**Published:** 2011-04-18

**Authors:** Ho Sik Rho, Amal Kumar Ghimeray, Dae Sung Yoo, Soo Mi Ahn, Sun Sang Kwon, Keun Ha Lee, Dong Ha Cho, Jae Youl Cho

**Affiliations:** 1 R & D Center, AmorePacific Corporation, Yongin, Korea; 2 College of Biomedical Science, Kangwon National University, Chuncheon, Korea; 3 Skin Biotechnology Center, Kyung Hee University, Suwon, Korea; 4 R & D Center, Morechem Corporation, Seoul, Korea; 5 Department of Genetic Engineering, Sungkyunkwan University, Suwon, Korea

**Keywords:** kaempferol, rhamnoside, depigmentation, anti-inflammation

## Abstract

The objective of this study was to examine the biological activity of kaempferol and its rhamnosides. We isolated kaempferol (**1**), α-rhamnoisorobin (**2**), afzelin (**3**), and kaempferitrin (**4**) as pure compounds by far-infrared (FIR) irradiation of kenaf (*Hibiscus cannabinus* L.) leaves. The depigmenting and anti-inflammatory activity of the compounds was evaluated by analyzing their structure-activity relationships. The order of the inhibitory activity with regard to depigmentation and nitric oxide (NO) production was kaempferol (**1**) > α-rhamnoisorobin (**2**) > afzelin (**3**) > kaempferitrin (**4**). However, α-rhamnoisorobin (**2**) was more potent than kaempferol (**1**) in NF-κB-mediated luciferase assays. From these results, we conclude that the 3-hydroxyl group of kaempferol is an important pharmacophore and that additional rhamnose moieties affect the biological activity negatively.

## 1. Introduction

**Figure 1:**
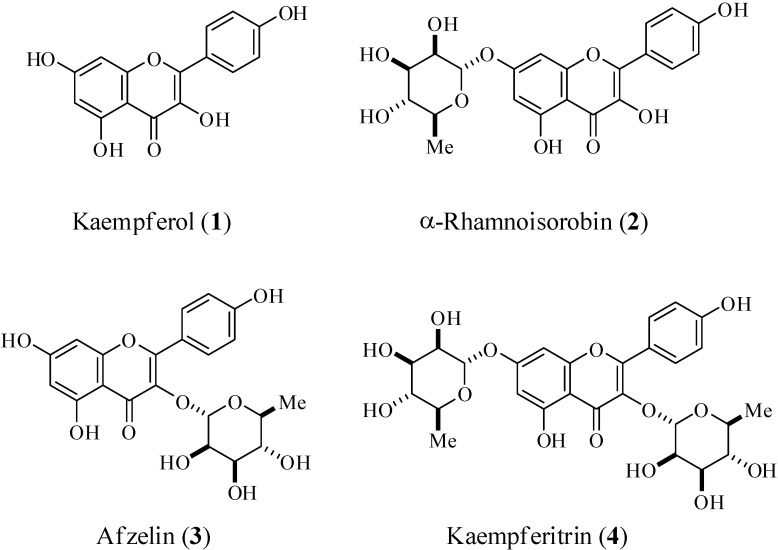
Structure of kaempferol and its rhamnosides.

Flavonoids are naturally occurring phenolic phytochemicals which are reported to possess various biologically important properties [1]. Consequently, the structure-activity relationships (SAR) of flavonoids have received much attention [2]. Despite the similarities among the flavonoid structures, minor structural modifications cause significant variations in their biological properties, with the number and specific positions of the hydroxyl groups determining the type and intensity of their activity. Generally, flavonoid glycosides are more abundant than flavonoids in natural products [3]. However, the SARs of flavonoid glycosides have not been fully studied [4]. Kenaf (*Hibiscus cannabinus* L.) is a member of the Malvaceae family and is used as a traditional folk medicine in Africa and India. It exhibits a broad spectrum of biological properties such as hepatoprotective activity [5], anti-oxidative activity [6], haematinic activity [7], and immunomodulatory effects [8]. Recently, we identified kaempferitrin (kaempferol-3,7-*O*-α-dirhamnoside) as one of the main components in kenaf leaf extracts. Far-infrared (FIR) irradiation dry kenaf leaf powder showed derhamnosylation products. Recently, the derhamnosylation products were isolated as pure compounds [9]. Chemical investigations identified their structures as kaempferol, α-rhamnoisorobin (kaempferol 7-*O*-α-L-rhamnopyranoside), and afzelin (kaempferol 3-*O*-α-L-rhamnopyranoside) (Figure 1). In this study, we evaluated the depigmenting and anti-inflammatory properties of kaempferol and its rhamnosides by analyzing their structure-activity relationships.

## 2. Results and Discussion

### 2.1. Depigmenting activity

We previously reported the anti-tyrosinase activities of kaempferol and its rhamnosides [9]. Compounds **1** (kaempferol) and **2** (α-rhamnoisorobin) showed tyrosinase inhibitory activities. However, compounds **3** (afzelin) and **4** (kaempferitrin) showed no inhibitory activities. In this study, we investigated the relationship of structure and biological activity in these compounds by evaluating the inhibitory activity of kaempferol and its rhamnosides on melanogenesis and inflammation. The depigmenting activity and cytotoxicity are summarized in Table 1.

**Table 1:** Depigmenting activities of kaempferol and its rhamnosides.

Compound	Inhibitory activity^a^ [IC_50_, (μM)]		
	Tyrosinase	Depigmentation	Cytotoxicity
Arbutin	330.2 ± 1.1	170.8 ± 2.1	>200
Kaempferol (**1**)	171.4 ± 0.9	37.66 ± 0.1	25.6 ± 0.9
α-Rhamnoisorobin (**2**)	>400 (39.1 %)^b^	39.45 ± 0.4	22.9 ± 1.3
Afzelin (**3**)	>400	>100	>100
Kaempferitrin (**4**)	>400	>100	>100

^a^ Values were determined from logarithmic concentration-inhibition curves and are the means of three experiments. ^b^ Inhibitory activity (% of control) at 400 μM.

We used B16 melanoma cells to evaluate the depigmenting activity [10]. Arbutin was used as the positive control. Compounds **1** (kaempferol) and **2** (α-rhamnoisorobin) showed inhibitory activity. However, their activity may originate from their cytotoxicity, as **1** and **2** showed cytotoxicity towards B16 melanoma cells. Compounds **3** (afzelin) and **4** (kaempferitrin) showed neither inhibitory activity nor cytotoxicity. These results suggest that the 3-hydroxyl group of kaemferol is a pharmacophore for depigmenting activity and cytotoxicity.

### 2.2. Anti-inflammatory activity

Next, we evaluated the anti-inflammatory activity by measuring both the inhibition of the nitric oxide (NO) production [11] and the suppression of the NF-κB-mediated luciferase assays [12]. We estimated the inhibitory activity of kaempferol and its rhamnosides relative to NO production, induced by LPS in RAW264.7 cells, and the NF-κB-mediated luciferase activity relative to a non-specific phosphodiesterase inhibitor such as pentoxifylline and BAY11-7082, an IκBα kinase inhibitor, as positive controls. Table 2 shows the inhibitory activity of kaempferol and its rhamnosides against NO production.

**Table 2:** NO inhibitory activity of kaempferol and its rhamnosides.

Compound	Inhibitory activity^a^ [IC_50_, (μM)]	
	NO	Cytotoxicity
Pentoxifylline	446.0 ± 1.1	>1000
Kaempferol (**1**)	15.4 ± 0.2	>100
α-Rhamnoisorobin (**2**)	37.7 ± 2.0	>100
Afzelin (**3**)	>100 (98.3 %)^b^	>100
Kaempferitrin (**4**)	>100 (108.1 %)^b^	>100

^a^ Values were determined from logarithmic concentration-inhibition curves and are the means of three experiments. ^b^ Inhibitory activity (% of control) at 100 μM.

The inhibitory activity for NO production is very similar to that of tyrosinase inhibition. The NO inhibitory activity decreased in the order of kaempferol (**1**), α-rhamnoisorobin (**2**), afzelin (**3**), and kaempferitrin (**4**). Compounds **1** (IC_50_ = 15.4 μM) and **2** (IC_50_ = 37.7 μM) showed potent inhibitory activity without cytotoxicity. An additional rhamnose moiety at the 3-position caused a loss in NO inhibitory activity. The effect of kaempferol and its glycosides on the NF-κB-mediated luciferase activity was also evaluated (Table 3).

**Table 3:** Effects of kaempferol and its glycosides on the NF-κB-mediated luciferase activity.

Compound	Inhibitory activity^a^ [IC_50_, (μM)]
BAY11-7082	11.5 ± 0.1
Kaempferol (**1**)	90.3 ± 5.1
α-Rhamnoisorobin (**2**)	36.2 ± 3.3
Afzelin (**3**)	>100
Kaempferitrin (**4**)	>100

^a^ Values were determined from logarithmic concentration-inhibition curves and are the means of three experiments.

Kaempferol (**1**) and α-rhamnoisorobin (**2**) also inhibited the NF-κB-mediated luciferase activities. However, the activity order of the two compounds was different. Compound **2** (IC_50_ = 36.2 μM) was more potent than compound **1** (IC_50_ = 90.3 μM). 

### 2.3. Expression of iNOS mRNA

To elucidate the mechanism underlying the inhibition of the NO production, we examined the effectof kaempferol (**1**) and α-rhamnoisorobin (**2**) on the expression of iNOS mRNA in LPS-activated RAW 264.7 cells [13]. It is well known that iNOS facilitates the synthesis of NO, and the transcriptional factor of NF-κB indirectly causes suppression of the NO production by affecting the iNOS expression [14]. 

**Figure 2:**
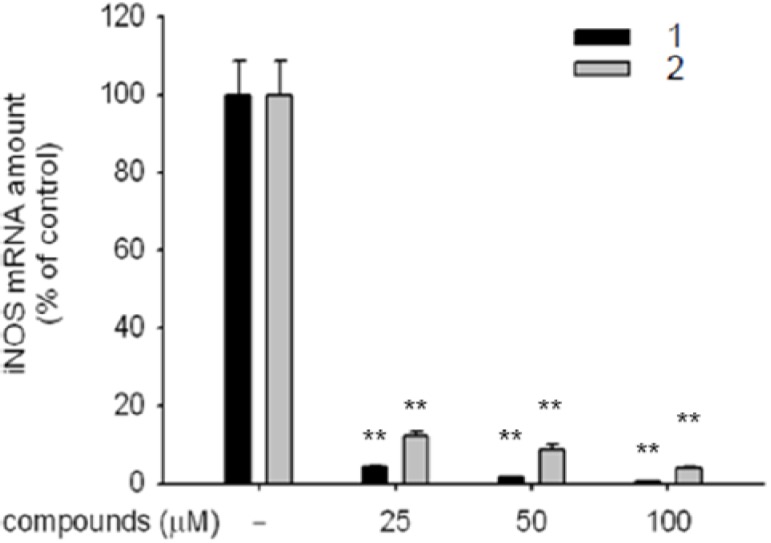
Effects of kaempferol (**1**) and α-rhamnoisorobin (**2)** on the expression of iNOS mRNA in LPS-activated macrophages. **: p <0.01 compared to control.

RAW 264.7 cells were treated with different concentrations of compound **1** and **2** (25–100 μM) and then stimulated with LPS (1 μg/mL) for 6 h. The treatment with kaempferol (**1**) and α-rhamnoisorobin (**2**) suppressed the expression of iNOS mRNA in a dose-dependent manner (Figure 2). These results suggested that the inhibition of NO production by compound **1** and **2** was caused by the suppression of the iNOS mRNA. The effect of kaempferol (**1**) on iNOS mRNA expression was more potent than that of α-rhamnoisorobin (**2**).

## 3. Experimental

### 3.1. Measurements of melanin content

B16 cells (5 × 10^4^ cells/well) were incubated with various concentrations of kaempferol and its rhamnosides or arbutin for 5 days. The cell pellets were then dissolved in 1 N NaOH in 10% DMSO (500 μL) at 80 °C for 1 h. The relative melanin content was determined by measuring the absorbance at 475 nm with an ELISA reader (Molecular Devices Corp., Menlo Park, CA, USA). A standard synthetic melanin curve (0–500 μg⁄mL) was prepared in triplicate for each experiment.

### 3.2. Measurements of NO production

RAW264.7 cells (1 × 10^6^ cells/mL) were preincubated with kaempferol and its rhamnosides or arbutin for 30 min and continuously activated with LPS (1 μg/mL) for 24 h. The nitrite in the culture supernatants was measured by adding Griess reagent (100 μL, 1% sulfanilamide and 0.1% *N*-[1-naphthyl]-ethylenediamine dihydrochloride in 5% phosphoric acid) to samples of the medium (100 μL) for 10 min at room temperature. The OD at 570 nm (OD_570_) was measured using a Spectramax 250 microplate reader (Molecular Devices). A standard curve of NO was made with sodium nitrite.

### 3.3. Measurements of cytotoxicity

After the preincubation of RAW264.7 cells (1 × 10^6^ cells/mL) for 18 h, kaempferol and its rhamnosides or arbutin (0–100 μM) were added to the cells and incubated for 24 h. The cytotoxic effect of kojyl thioether derivatives was then evaluated by a conventional MTT assay. At 3 h prior to culture termination, MTT solution (10 μL, 5 mg/mL in a phosphate buffered-saline, pH 7.4) was added and the cells were continuously cultured until termination. The incubation was halted by the addition of 15% sodium dodecyl sulfate into each well, solubilizing formazan. The absorbance at 570 nm (OD_570–630_) was measured by a Spectramax 250 microplate reader.

### 3.4. Luciferase assay

HEK293 cells (1 × 10^6^ cells/mL) were transfected with plasmids containing NF-κB-Luc, (1 μg/mL each) as well as β-galactosidase (0.5 μg/mL) using the PEI method in a 12-well plate for 48 h, and they were treated with testing compounds in the presence or absence of PMA (0.1 μM) for 24 h. Luciferase assay was performed using the Luciferse Assay System (Promega).

### 3.5. mRNA detection by quantitative and semi-quantitative real-time reverse transcription-PCR

The total RNA from the LPS treated-RAW264.7 cells was prepared by adding TRIzol Reagent (Gibco BRL), as per the manufacturer’s instructions. Real-time PCR reactions were conducted using MuLV reverse transcriptase. The primers (Bioneer, Daejeon, Korea) were used as previously reported [15].

### 3.6. Statistical analysis

Student’s *t*-test and one–way ANOVA were used to determine the statistical significance. Data (Table 1, Table 2, and Table 3, and Figure 2) expressed as means ± standard errors (SEM) were taken from at least three independent experiments performed in triplicate. *P* < 0.05 was considered statistically significant.

## 4. Conclusions

In the present study, we have evaluated the biological activity of kaempferol (**1**) and some of its rhamnosides such as α-rhamnoisorobin (**2**), afzelin (**3**), and kaempferitrin (**4**). Depigmenting and anti-inflammatory activities such NO production and NF-κB-mediated luciferase activity were tested. In all tested assays, kaempferol (**1**) and α-rhamnoisorobin (**2**) showed inhibitory activity. These results imply that the 3-hydroxyl group of kaempferol is an important pharmacophore and that additional rhamnose moieties at 3 of kaempferol have a negative effect on the biological activity. RT-PCR analysis suggested that compound **1** and **2** inhibited the NO production by suppressing the iNOS mRNA expression.

## References

[1] Hollman P.C., Katan M.B. (1999). Health effects and bioavailability of dietary flavonoids. Free Radic. Res..

[2] Arora A., Nair M.G., Strasburg G.M. (1998). Structure-activity relationships for antioxidant activities of a series of flavonoids in a liposomal system. Free Radic. Biol. Med..

[3] Yen C.T., Hsieh P.W., Hwang T.L., Lan Y.H., Chang F.R., Wu Y.C. (2009). Flavonol glycosides from *Muehlenbeckia platyclada* and their anti-inflammatory activity. Chem. Pharm. Bull..

[4] De Melo G.O., Malvar D.C., Vanderlinde F.A., Rocha F.F., Pires P.A., Costa E.A., De Matos L.G., Kaiser C.R., Costa S.S. (2009). Antinociceptive and anti-inflammatory kaempferol glycosides from *Sedum dendroideum*. J. Ethnopharmacol..

[5] Agbor G.A., Oben J.E., Nkegoum B., Takala J.P., Ngogang J.Y. (2005). Hepatoprotective activity of *Hibiscus cannabinus* (Linn.) against carbon tetrachloride and paracetamol induced liver damage in rats. Pak. J. Biol. Sci..

[6] Agbor G.A., Oben J.E., Ngogang J.Y., Xinxing C., Vinson J.A. (2005). Antioxidant capacity of some herb/spices from Cameroon: A comparative study of two methods. J. Agric. Food Chem..

[7] Agbor G.A., Oben J.E., Ngogang J.Y. (2005). Haematinic activity of *Hibiscus cannabinus*. Afr. J. Biotechnol..

[8] Lee Y.G., Byeon S.E., Kim J.Y., Lee J.Y., Rhee M.H., Hong S., Wu J.C., Lee H.S., Kim M.J., Cho D.H., Cho J.Y. (2007). Immunomodulatory effect of *Hibiscus cannabinus* extract on macrophage functions. J. Ethnopharmacol..

[9] Rho H.S., Ahn S.M., Lee B.C., Kim M.K., Ghimeray A.K., Jin C.W., Cho D.H. (2010). Changes in flavonoid content and tyrosinase inhibitory activity in kenaf leaf extract after far-infrared treatment. Bioorg. Med. Chem. Lett..

[10] Liu S.-H., Pan I.-H., Chu I.-M. (2007). Inhibitory effect of *p*-Hydroxybenzyl alcohol on tyrosinase activity and melanogenesis. Biol. Pharm. Bull..

[11] Rho H.S., Yoo D.S., Ahn S.M., Kim M.K., Cho D.H., Cho J.Y. (2010). Inhibitory activities of kojyl thioether derivatives against nitric oxide production by lipopolysaccharide. Bull. Korean Chem. Soc..

[12] Jeon S.J., Kwon K.J., Shin S., Lee S.H., Rhee S.Y., Han S.H., Lee J., Kim H.Y., Cheong J.H., Ryu J.H., Min B.S., Ko K.H., Shin C.Y. (2009). Inhibitory effects of *Coptis japonica* alkaloids on the LPS-induced activation of BV2 microglia cells. Biomol. Ther..

[13] Yuan H.D., Kim S.J., Quan H.Y., Huang B., Chung S.H. (2010). Ginseng leaf extract prevents high fat diet-induced hyperglycemia and hyperlipidemia through AMPK activation. J. Ginseng Res..

[14] Kleinert H., Schwarz P.M., Forstermann U. (2003). Regulation of the expression of inducible nitric oxide synthase. Biol. Chem..

[15] Kim J.Y., Lee Y.G., Kim M.Y., Byeon S.E., Rhee M.H., Park J., Katz D.R., Chain B.M., Cho J.Y. (2010). Src-mediated regulation of inflammatory response by actin polymerization. Biochem. Pharmacol..

